# tPA Deficiency in Mice Leads to Rearrangement in the Cerebrovascular Tree and Cerebroventricular Malformations

**DOI:** 10.3389/fncel.2015.00456

**Published:** 2015-11-30

**Authors:** Christina Stefanitsch, Anna-Lisa E. Lawrence, Anna Olverling, Ingrid Nilsson, Linda Fredriksson

**Affiliations:** ^1^Department of Medical Biochemistry and Biophysics, Division of Vascular Biology, Karolinska InstitutetStockholm, Sweden; ^2^Department of Internal Medicine, Division of Cardiovascular Medicine, University of Michigan Medical SchoolAnn Arbor, MI, USA

**Keywords:** tPA, PDGF, blood-brain barrier, neurovascular unit, neurovascular coupling, lateral ventricles, ERG, vascular permeability

## Abstract

The serine protease tissue-type plasminogen activator (tPA) is used as a thrombolytic agent in the management of ischemic stroke, but concerns for hemorrhagic conversion greatly limits the number of patients that receive this treatment. It has been suggested that the bleeding complications associated with thrombolytic tPA may be due to unanticipated roles of tPA in the brain. Recent work has suggested tPA regulation of neurovascular barrier integrity, mediated via platelet derived growth factor (PDGF)-C/PDGF receptor-α (PDGFRα) signaling, as a possible molecular mechanism affecting the outcome of stroke. To better understand the role of tPA in neurovascular regulation we conducted a detailed analysis of the cerebrovasculature in brains from adult tPA deficient (*tPA^−/−^*) mice. Our analysis demonstrates that life-long deficiency of tPA is associated with rearrangements in the cerebrovascular tree, including a reduction in the number of vascular smooth-muscle cell covered, large diameter, vessels and a decrease in vessel-associated PDGFRα expression as compared to wild-type (WT) littermate controls. In addition, we found that ablation of tPA results in an increased number of ERG-positive endothelial cells and increased junctional localization of the tight junction protein ZO1. This is intriguing since ERG is an endothelial transcription factor implicated in regulation of vascular integrity. Based on these results, we propose that the protection of barrier properties seen utilizing these *tPA*^−/−^ mice might be due, at least in part, to these cerebrovascular rearrangements. In addition, we found that *tPA*^−/−^ mice displayed mild cerebral ventricular malformations, a feature previously associated with ablation of PDGF-C, thereby providing an *in vivo* link between tPA and PDGF signaling in central nervous system (CNS) development. Taken together, the data presented here will advance our understanding of the role of tPA within the CNS and in regulation of cerebrovascular permeability.

## Introduction

The serine protease tissue-type plasminogen activator (tPA) is primarily known for its role in fibrinolysis via proteolytic activation of plasminogen into plasmin (Collen, 2001). The observation that tPA directly binds to fibrin (van Zonneveld et al., 1986; Verheijen et al., 1986) and thereby facilitates localized generation of plasmin has led to the use of tPA as a thrombolytic agent for treatment of acute myocardial infarction and ischemic stroke (Collen, 2001). In fact, tPA administration is the only FDA-approved thrombolytic therapy for acute ischemic stroke (The national institute of neurological disorders and stroke rt-PA stroke study group, 1995; Su et al., 2009). The use of thrombolytic tPA in ischemic stroke is however markedly limited due to concerns for hemorrhagic complications and the requirement that it is administered within a few hours of onset of symptoms (The national institute of neurological disorders and stroke rt-PA stroke study group, 1995; Ahmed et al., 2010). The mechanism by which thrombolytic tPA might lead to hemorrhagic transformation of ischemic stroke is not completely understood, but it appears to be due in part to unique activities that tPA has in the brain beyond its well established role in fibrinolysis (Nicole et al., 2001; Wang et al., 2003; Yepes et al., 2003; Su et al., 2009).

The role of tPA within the central nervous system (CNS) is controversial (Su et al., 2009; Yepes et al., 2009; Lemarchant et al., 2012; Schielke and Lawrence, 2012). It has been proposed that tPA directly affects multiple processes, including neuronal development/plasticity/excitotoxicity (Tsirka et al., 1996; Seeds et al., 2003; Li et al., 2013), microglial activation (Tsirka et al., 1997; Rogove and Tsirka, 1998), as well as regulation of cerebrovascular permeability (Yepes et al., 2003; Su et al., 2008). In a recent paper, we proposed that the neurovascular events regulated by tPA might provide a unifying pathway for many of these pleotropic effects of tPA in the CNS (Fredriksson et al., 2015). We argued that tPA-induced changes in cerebrovascular permeability might lead to a loss of precise control of the extracellular environment, which in turn, might promote dysregulation of neuronal signaling pathways (Fredriksson et al., 2015).

To better understand the role of tPA in cerebrovascular regulation we have conducted a detailed analysis of the cerebrovasculature and the neurovascular unit in brains from adult tPA deficient (*tPA^−/−^*) mice. We demonstrate that tPA deficiency is associated with abnormal cerebral vascularization, including a reduction in the amount of large diameter vessels. In addition, we found a significant reduction of platelet derived growth factor receptor-α (PDGFRα) expression around cerebral vessels of *tPA*^−/−^ mice which is particularly interesting given our previous findings showing that the effect of tPA in regulation of cerebrovascular permeability is mediated via activation of platelet derived growth factor (PDGF)-C/PDGFRα signaling on perivascular astrocytes (Fredriksson et al., 2004, 2005; Su et al., 2008). Intriguingly, we also found that ablation of tPA results in increased number of ERG-positive endothelial cells. ERG (ETS related gene) is a member of the ETS family of transcription factors implicated in vascular development (reviewed in Randi et al., 2009) and overexpression of ERG in vivo was recently shown to reduce vascular permeability (Birdsey et al., 2015). In addition, we found that *tPA*^−/−^ mice displayed mild cerebral ventricular malformations, a feature previously associated with ablation of PDGF-C (Fredriksson et al., 2012), thereby providing an *in vivo* link between tPA and PDGF signaling in CNS development. These findings will aid in our understanding of the role of tPA in the CNS.

## Materials and Methods

### Animal Strains

Age- and gender-matched tPA deficient mice (*tPA*^−/−^; Carmeliet et al., 1994), back-crossed at least 10 generations into C57BL/6J background, and their wild-type (WT) littermate controls obtained from heterozygous breedings were used. All animal experiments were approved by the local committee for animal experiments at the University of Michigan, USA, and the studies were conducted in accordance with the United States Public Health Service’s Policy on Humane Care and Use of Laboratory Animals.

### Immunostaining and Confocal Microscopy

Tissue preparation for sectioning and immunostaining were conducted using standard protocols. Mice were anesthetized with isoflurane and following transcardial perfusion fixation with 4% paraformaldehyde (PFA) in phosphate buffered saline (PBS) the brains were removed and postfixed in 4% PFA 1 h at room temperature (RT) and then kept in 30% sucrose, 4°C overnight.

For immunofluorescence, vibratome sections (50 μm) were cut and stained free floating in 24-well plates. The sections were permeabilized and blocked with 1% bovine serum albumin (BSA) in 0.5% TritonX-100/PBS overnight at 4°C, followed by incubation with primary antibodies in blocking solution overnight at 4°C (1:200 dilution). After thorough washes the sections were incubated with fluorescent-conjugated secondary antibodies overnight at 4°C. The specific primary antibodies used were: aquaporin 4 (AB2218; Millipore, Billerica, MA, USA), α-smooth muscle actin (ASMA)-Cy3 (C6198; Sigma-Aldrich, St. Louis, MO, USA), CD13 (MCA2183; AbD Serotec, Oxford, UK), CD31 (553370; BD Biosciences, Franklin Lakes, NJ, USA), ERG1 (ab92513; Abcam Plc, Cambridge, UK), collagen IV (21501470; AbD Serotec), glial fibrillary acidic protein (GFAP; Z0334; Dako, Glostrup, Denmark), glucose transporter 1 (GLUT1; sc-1605; Santa Cruz Biotechnology, Santa Cruz, CA, USA), PDGFRα (AF1062; R&D Systems, Minneapolis, MN, USA), podocalyxin (AF1556; R&D Systems), and ZO1 (339100; Invitrogen, Molecular Probes). For S100B (Z0311; Dako) antigen retrieval was performed by boiling 10 min in target retrieval solution (S1700; Dako). Appropriate fluorophore-conjugated secondary antibodies were used for multi color detection (Alexa Fluor 488, 568 and 647; Life Technologies, Molecular Probes, Grand Island, NY, USA) and DAPI (4′,6-Diamidino-2-Phenylindole, Dihydrochloride, 0.2 μg/ml) was included in the last PBS wash to visualize the nuclei. The sections were then mounted on Superfrost Plus slides (Thermo Fisher Scientific Inc., Waltham, MA, USA) with ProLong Gold Antifade reagent (P36930; Life Technologies). All stainings were repeated two-four independent times.

All images were acquired at RT with a Zeiss LSM700 confocal microscope or a Zeiss Axio Observer Z1 inverted microscope and the ZEN 2009 software (Carl Zeiss Microimaging GmbH, Jena, Germany). Stained brain sections from *tPA*^−/−^ mice (*n* = 5) and WT littermates (*n* = 5) were analyzed by two independent investigators blinded to the study group. In addition, as a control of the quantification at least one set of images from each respective staining was reanalyzed by a second blinded investigator. *n* indicates the number of individual mice used in the study. The individual observations are based on analysis of four-eleven fields of view (same number of images per animal and settings for each respective staining within an individual experiment). The fields of view were taken in comparable anatomic positions in each animal and the anatomic position to be imaged was identified using the DAPI channel. The anatomic positions analyzed were from brain regions where high levels of tPA expression has been reported, including cortex, hippocampus and amygdala (Yu et al., 2001). We did not find any evidence of sub-regional effects or differences during our analysis. The images were processed and analyzed using Volocity 3D image analysis software (PerkinElmer, Waltham, MA, USA), Photoshop CS5 (Adobe, San Jose, CA, USA) or ImageJ64 (National Institutes of Health, Bethesda, MD, USA). For quantification of antibody immunoreactivity using intensity, all images were acquired using the same settings (within the respective staining experiment) and the number of pixels above a set threshold was determined. Each field of view analyzed was from a maximum intensity *Z*-stack image (15–22 μm stack) or from an epifluorescent image. The result from all the fields of view in a given animal was averaged to obtain the value for that individual. Individual values and group mean ± SEM are shown. The images shown are representative of the respective staining. Detailed information regarding all the stainings and analysis performed is summarized in the supplementary table 1 (Table S1). Brightness and contrast settings were changed to generate the final image and were applied equally to the entire image and within the same set of images.

### Magnetic Resonance Imaging Scans

All magnetic resonance imaging scans (MRI) were conducted at the Center for Molecular Imaging, Department of Radiology, University of Michigan. During the imaging procedure the mice were anesthetized with isoflurane and the body temperature was maintained using forced heated air. The MRI examinations were performed in a 7.0T Varian MR scanner (183-mm horizontal bore, Varian, Palo Alto, CA, USA). A double-tuned volume radiofrequency coil was used to scan the head region of the mice. Coronal T2-weighted images were acquired using a fast spin-echo sequence with the following parameters: repetition time (TR)/effective echo time (TE), 4000/60 ms; echo spacing, 15 ms; number of echoes, 8; field of view (FOV), 20×20 mm; matrix, 256×128; slice thickness, 0.5 mm; number of slices, 25; and number of scans, 1 (total scan time ~2 min.). Photoshop and Image J were used to assess differences in ventricular area and the analysis was conducted blinded.

### Statistical Analysis

Statistical analysis was performed using GraphPad Prism 6.0 statistical software (GraphPad Software, La Jolla, CA, USA). Statistical significance was determined by student’s unpaired *t*-test and *P* values less than 0.05 were considered statistically significant and are indicated in the figures by asterisks.

## Results

### tPA Deficiency Affects Cerebral Vessel Size

**Figure 1:**
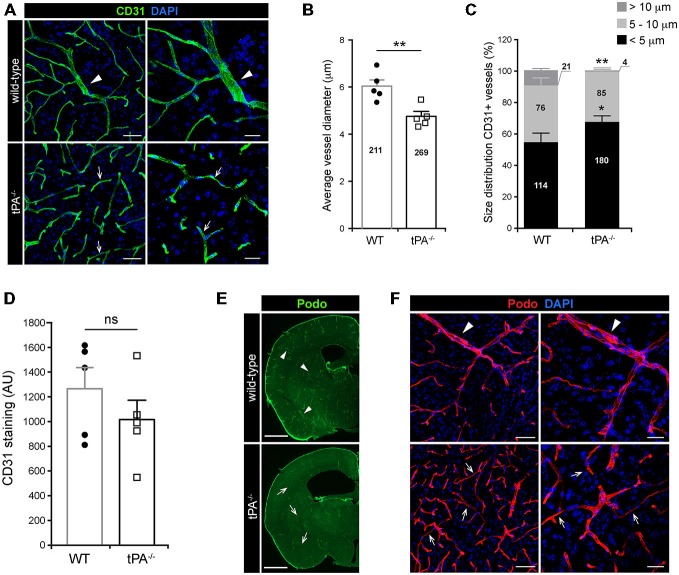
**Decreased number of large diameter vessels in tPA^−/−^ brains.** Immunofluorescent staining of murine brain sections from *tPA^−/−^* deficient mice *n* = 5) and wild-type (WT) littermate controls (WT, *n* = 5) with **(A–D)** the endothelial cell marker CD31 and **(E–F)** podocalyxin (Podo) showed fewer large diameter vessels (arrowheads) and increased number of small diameter vessels (arrows) in *tPA*^−/−^ mice compared to WT littermate controls. Quantification of the CD31 staining from four confocal *Z*-stacks per animal revealed that the average vessel diameter was significantly lower in *tPA*^−/−^ brains as compared to WT littermate controls **(B)** and that there was a significant redistribution toward smaller diameter vessels in the cerebrovasculature of *tPA*^−/−^ mice **(C)**. The number of vessels analyzed is displayed on the respective bars. There was no significant difference in the overall amount of CD31 positive staining as quantified by pixel intensity **(D)**. Staining with podocalyxin confirmed the results seen with staining for CD31 **(E–F)**. The data shown are representative quantifications of four to nine maximum intensity confocal *Z*-stacks per animal from four independent staining experiments with CD31 and Podocalyxin, respectively. The images display **(A,F)** maximum intensity projections generated from confocal *Z*-stacks (22 μm) and **(E)** stitched tiling of epifluorescent images (taken with 10× objective). Cell nuclei were visualized with DAPI. Data presented as mean ± SEM. Statistical significance was determined by student’s unpaired *t*-test. *P < 0.05; **P < 0.01;* ns* = non significant relative to control. Scale bars **(A,F)** left panels, 50 μm; right panels, 20 μm, **(E)** 1 mm. Arbitrary units, A.U.

A growing body of evidence is showing that tPA is both necessary and sufficient to regulate cerebrovascular permeability (Yepes et al., 2003; Su et al., 2008; Fredriksson et al., 2015). In order to gain a better understanding of the role of tPA in controlling cerebrovascular events we performed a thorough analysis of the vascular bed in brain sections from tPA deficient (*tPA^−/−^*) mice. Immunofluorescent stainings using CD31 antibodies, a marker for vascular endothelial cells, revealed an abnormal vascularization in the brains of adult *tPA*^−/−^ mice (*n* = 5) compared to littermate WT controls (*n* = 5; Figure 1A). In *tPA*^−/−^ brains the vascular bed showed an apparent decrease in large diameter vessels (arrowheads) and increase in small diameter vessels (arrows) relative to WT mice (Figure 1A). This was confirmed by quantification of the CD31 stainings, showing a significant decrease (*P* < 0.01) in vessel diameter in *tPA*^−/−^ mice (4.75 ± 0.2 μm) as compared to WT littermate controls (6.03 ± 0.3 μm; Figure 1B). This decrease in average vessel size in *tPA*^−/−^ mice was due to a significant reorganization of the vascular bed in *tPA*^−/−^ brains relative to WT littermate controls, with increased number of small diameter vessels (<5 μm; WT = 54 ± 6% vs. *tPA*^−/−^ = 67 ± 4%, *P* < 0.05) and fewer large diameter vessels (>10 μm; WT = 10 ± 1% vs. *tPA*^−/−^ = 1 ± 0.8%, *P* < 0.01; Figure 1C). This was accompanied with an overall, but non-significant (*P* = 0.31), reduction in the total amount of CD31 staining in *tPA*^−/−^ brains (80 ± 12% of WT) compared to WT littermate controls (Figure 1D). Staining with podocalyxin antibodies, another marker of vascular endothelial cells, confirmed the loss of large diameter vessels (arrowheads) and increase in small diameter vessels (arrows) in *tPA*^−/−^ brains relative to WT littermate controls (Figures 1E,F).

### tPA Deficiency is Associated with Increased Number of ERG-Positive Endothelial Cells and Enhanced Junctional Localization of ZO1 in the Murine Brain

**Figure 2:**
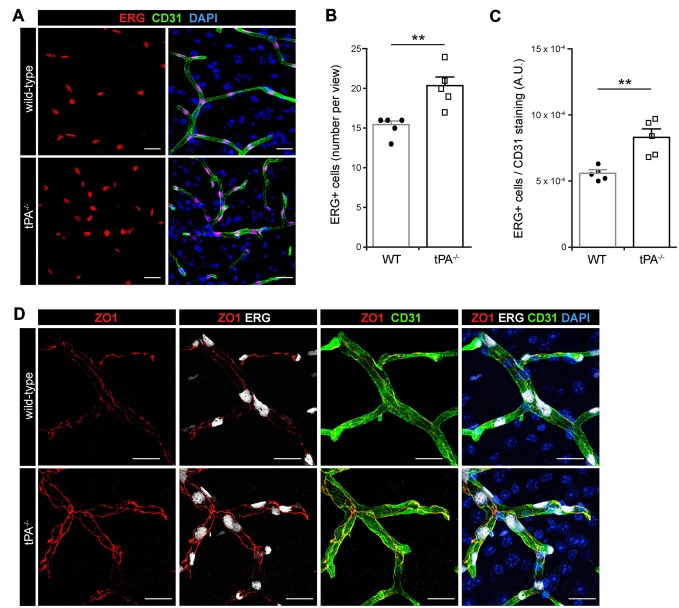
**Increased number of ERG+ endothelial cells and enhanced tight junction staining in tPA^−/−^ mice. (A)** Immunofluorescent stainings of murine brain sections from *tPA^−/−^* mice (*n* = 5) and WT littermate controls (WT, *n* = 5) with antibodies for the endothelial transcription factor ERG showed what appeared to be increased number of endothelial cells in *tPA*^−/−^ mice as compared to WT littermate controls. **(B–C)** This was confirmed through quantification of the number of ERG+ nuclei from four maximum intensity confocal *Z*-stacks per animal. This revealed that there was significantly more ERG-positive cells in *tPA*^−/−^ brains compared to WT littermate controls **(B)**, also when normalized to CD31 staining as quantified by pixel intensity (arbitrary units, A.U.) **(C)**. The data shown are representative quantifications of four to nine maximum intensity confocal *Z*-stacks per animal from two independent staining experiments. **(D)** Staining with antibodies against the tight junction protein ZO1 illustrated increased junctional expression in cerebral vessels in *tPA*^−/−^ mice as compared to WT littermate controls. The images display maximum intensity projections generated from confocal *Z*-stacks (22 μm). Data presented as mean ± SEM. Statistical significance was determined by student’s unpaired *t*-test and **P < 0.01 relative to control. Scale bars **(A,D)** 20 μm.

In order to characterize whether the cerebrovascular changes seen in *tPA^−/−^* mice were associated with altered number of endothelial cells we conducted immunofluorescent stainings using antibodies against the endothelial transcription factor ERG. Unexpectedly, we found an apparent increase in the number of ERG-positive cells in the brains of *tPA*^−/−^ mice (*n* = 5) relative to WT littermate controls (*n* = 5; Figure 2A). This was confirmed by quantification of the number of ERG-positive cells, showing a significant (*P* < 0.01) overall increase in the *tPA*^−/−^ mice (20.2 ± 1.2 ERG+ cells/field of view) compared to WT littermate controls (15.2 ± 0.6 ERG+ cells/field of view; Figure 2B). To ensure that the increase of ERG-positive cells was not due to an increase in the number of small vessels in *tPA*^−/−^ brains, we normalized the number of ERG+ cells to the amount of CD31 staining. This verified that there were significantly more (*P* < 0.01) ERG+ cells in the cerebral vessels of *tPA*^−/−^ mice (149 ± 11% of WT; Figure 2C). This was particularly interesting since ERG has been implicated in vascular development, control of vascular permeability and maintenance of the integrity of the endothelial junctions (Randi et al., 2009; Birdsey et al., 2015). We therefore investigated the appearance of endothelial tight junctions in *tPA*^−/−^ mice by immunofluorescent stainings using antibodies against the tight junction protein ZO1. We found that ZO1 appeared to show an increased junctional localization in vessels in *tPA*^−/−^ brains compared to WT littermate controls (Figure 2D), suggesting that *tPA*^−/−^ mice might have tighter endothelial cell-cell junctions.

### tPA Deficiency Leads to a Redistribution in Vascular Mural Cell Coverage

**Figure 3:**
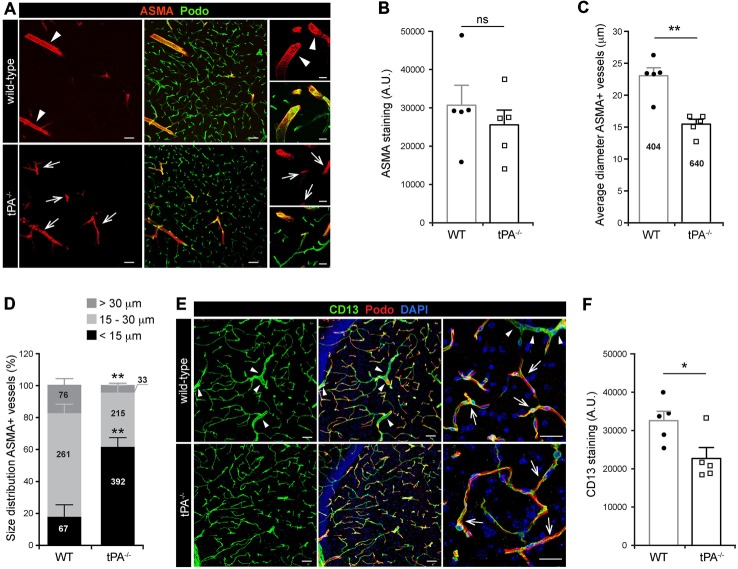
**Decreased number of large diameter ASMA+ vessels in tPA^−/−^ brains. (A)** Immunofluorescent staining of murine brain sections from mice *tPA^−/−^* (*n* = 5) and WT littermates (*n* = 5) with the vascular smooth muscle cell (vSMC) marker for alpha-smooth muscle actin (ASMA) displayed a redistribution of ASMA-positive (ASMA+) vessels in *tPA*^−/−^ mice compared to WT littermate controls. There appeared to be more ASMA+ vessels with smaller diameter (arrows) in *tPA*^−/−^ brains and fewer large ASMA+ diameter vessels (arrowheads) commonly seen in WT brains. **(B–D)** Quantification of the ASMA staining from eleven confocal images per animal revealed that there was no significant difference in the overall amount of ASMA staining as quantified by pixel intensity **(B)**, although the average vessel diameter was significantly lower in *tPA*^−/−^ brains as compared to WT littermate controls **(C)**. In addition, there was a significant redistribution toward smaller diameter ASMA+ vessels in the cerebrovasculature of *tPA*^−/−^ mice compared to WT littermate controls **(D)**. The number of vessels analyzed is displayed on the respective bars. The data shown are representative quantifications of four to eleven confocal images per animal from three independent staining experiments. **(E)** Staining with the vascular mural cell marker CD13, which stains pericytes, revealed that large diameter vessels positive for CD13, normally present in WT control brains (arrowheads), was essentially lost in *tPA*^−/−^ brains. However, the pericyte coverage of capillaries appeared normal (arrows). **(F)** The loss of large diameter CD13+ vessels resulted in a significant reduction in CD13 staining in *tPA*^−/−^ brains (*n* = 5) compared to WT littermate controls (*n* = 5). Quantification of CD13 pixel intensity was made from nine confocal *Z*-stacks per animal. The data shown are representative quantifications of four to nine maximum intensity confocal *Z*-stacks per animal from three independent staining experiments. The images display **(A,E)** maximum intensity projections generated from confocal *Z*-stacks (22 μm). Cell nuclei were visualized with DAPI and vessels with podocalyxin (Podo). Data presented as mean ± SEM. Statistical significance was determined by student’s unpaired *t*-test. *P < 0.05; **P < 0.01;* ns* = non significant relative to control. Scale bars **(A,E)** left and middle panels, 50 μm; right panels, 20 μm. Arbitrary units, A.U.

Mural cells on the cerebral vascular tree include vascular smooth muscle cells (vSMCs) and capillary pericytes. These cells are known to play important roles in cerebrovascular events, including maintenance of the blood-brain barrier (BBB; Armulik et al., 2010; Daneman et al., 2010) and in regulation of vessel diameter and blood flow (Kornfield and Newman, 2014; Hill et al., 2015). To assess the distribution of vascular mural cell coverage on cerebral vessels in *tPA^−/−^* mice we performed immunofluorescent stainings using antibodies against ASMA, a marker for vSMCs, and CD13, a marker for pericytes. The stainings showed an apparent shift in the size of ASMA+ vessels, with more ASMA+ small diameter vessels (arrows) and fewer ASMA+ large diameter vessels (arrowheads) in *tPA*^−/−^ brains (*n* = 5) compared to WT littermate controls (*n* = 5; Figure 3A). There was no significant difference in the overall amount of ASMA+ staining (*P* = 0.46; Figure 3B) but the average diameter of ASMA+ vessels was significantly smaller (*P* < 0.01) in *tPA*^−/−^ mice (15.4 ± 0.7 μm) as compared to WT littermate controls (22.9 ± 1.3 μm; Figure 3C). *tPA*^−/−^ mice displayed significantly increased numbers of ASMA+ small diameter vessels (<15 μm; WT = 17 ± 8% vs. *tPA*^−/−^ = 61 ± 6%, *P* < 0.01) and fewer large diameter vessels (>30 μm; WT = 19 ± 3% vs. *tPA*^−/−^ = 5 ± 1%, *P* < 0.01; Figure 3D). The microvascular capillaries in the adult murine brain of *tPA*^−/−^ mice appeared to have normal coverage of pericytes as visualized by immunostainings with CD13 antibodies (arrows, Figure 3E). However, CD13 antibodies also stain vSMC in larger vessels (arrowheads, Figure 3E) and these vessels are essentially lost in *tPA*^−/−^ mice, resulting in an overall reduction in CD13 staining relative to WT littermate controls (*P* < 0.05; Figure 3F).

### The Basement Membrane and Astrocyte Distribution is not Affected by tPA Deficiency

**Figure 4:**
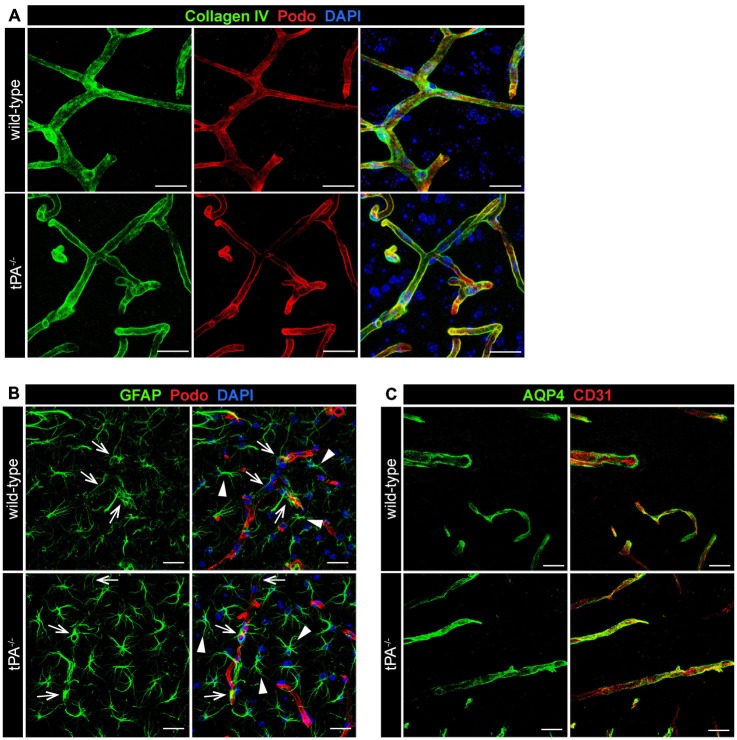
**Normal basement membrane and astrocyte distribution in tPA^−/−^ mice.** Immunofluorescent staining of murine brain sections from *tPA^−/−^* mice (*n* = 5) and WT littermate controls (*n* = 5) was conducted with antibodies directed against **(A)** collagen IV, a marker of the basement membrane, **(B)** glial fibrillary acidic protein (GFAP), a marker for astrocytes, and **(C)** aquaporin 4 (AQP4), a marker of perivascular astrocytic endfeet. These analyses revealed normal distribution of basement membrane **(A)** and perivascular astrocytes (arrows, **B,C)** around similar sized vessels in *tPA*^−/−^ mice and WT littermates. In addition, normal distribution of parenchymal astrocyte staining (arrowheads, **B**) was detected. The pictures are representative maximum intensity projections images generated from confocal *Z*-stacks from stainings on brain sections from WT and *tPA*^−/−^ mice and the stainings have been repeated at least two independent times. Cell nuclei were visualized with DAPI and vessels with podocalyxin (Podo) or CD31. Scale bars 20 μm.

To determine whether tPA deficiency affects the basement membrane and/or perivascular astrocyte distribution we analyzed sections from *tPA^−/−^* and WT littermate control brains stained with antibodies directed against collagen IV, a marker of the basement membrane; GFAP, a marker for astrocytes; and aquaporin 4 (AQP4), a marker of perivascular astrocytic endfeet. These analyses revealed what appeared as normal distribution of basement membrane around similar sized vessels in *tPA*^−/−^ mice (Figure 4A). Further, we detected similar distribution of GFAP+ astrocytes in the parenchyma (arrowheads) as well as GFAP+ perivascular astrocytes associated with comparable sized vessels (arrows) in the brains of *tPA*^−/−^ mice relative to WT littermate controls (Figure 4B). This was supported by stainings of astrocytic endfeet with AQP4 showing apparently normal perivascular astrocyte coverage in vessels of comparable size (Figure 4C).

### tPA Deficiency is Associated with Reduced Levels of Vessel-Associated PDGFRα Expression

**Figure 5:**
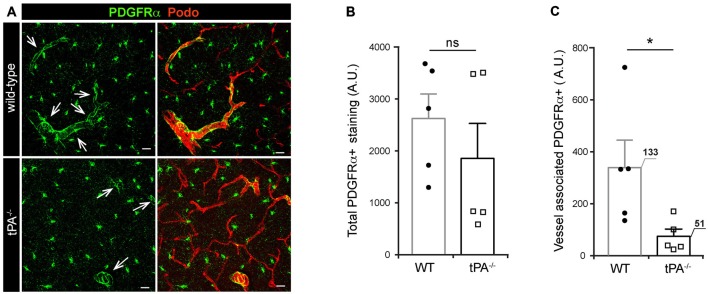
**Decreased expression of perivascular PDGFRα in brains of tPA^−/−^ mice. (A)** Immunofluorescent staining of murine brain sections from *tPA^−/−^* deficient mice (*n* = 5) and WT littermate controls (WT, *n* = 5) with antibodies directed against PDGFRα displayed fewer PDGFRα-positive (PDGFRα+) vessels (arrows) in *tPA*^−/−^ mice compared to WT littermate controls. **(B–C)** Quantification of the PDGFRα staining by pixel intensity from nine maximum intensity confocal *Z*-stacks per animal revealed that there was no significant difference in the overall amount of PDGFRα staining **(B)**, but that perivascular PDGFRα expression was significantly reduced in *tPA*^−/−^ mice as compared to WT littermate controls **(C)**. The number of PDGFRα+ vessels analyzed is displayed on the respective bars. The data shown are representative quantifications of nine maximum intensity confocal *Z*-stacks per animal and images from epifluorescent tiles of the entire brain from three independent staining experiments. The images display maximum intensity projections generated from confocal *Z*-stacks (22 μm). Cell nuclei were visualized with DAPI and vessels with podocalyxin (Podo). Data presented as mean ± SEM. Statistical significance was determined by student’s unpaired *t*-test and *P < 0.05;* ns* = non significant relative to control. Scale bars 20 μm. Arbitrary units, A.U.

Due to our previous findings showing that the effect of tPA in regulation of cerebrovascular permeability is mediated via activation of PDGFRα signaling on perivascular astrocytes in the neurovascular unit (Fredriksson et al., 2004, 2005, 2015; Su et al., 2008) we investigated the expression of PDGFRα in *tPA^−/−^* brains. Immunofluorescent stainings for PDGFRα displayed a reduction of PDGFRα expression around vessels (arrows) in *tPA*^−/−^ mice compared to WT littermate controls (Figure 5A). Quantification of the total amount and vessel-associated PDGFRα expression revealed that there was no significant difference (*P* = 0.38) in the total amount of PDGFRα+ staining between *tPA*^−/−^ mice and WT littermate controls (Figure 5B). Perivascular PDGFRα expression was on the other hand significantly reduced in *tPA*^−/−^ mice (21 ± 8% of WT) as compared to WT littermate controls (*P* < 0.05; Figure 5C).

### Asymmetry of the Cerebral Lateral Ventricles and Distorted Ependymal Lining in tPA Deficient Mice

**Figure 6:**
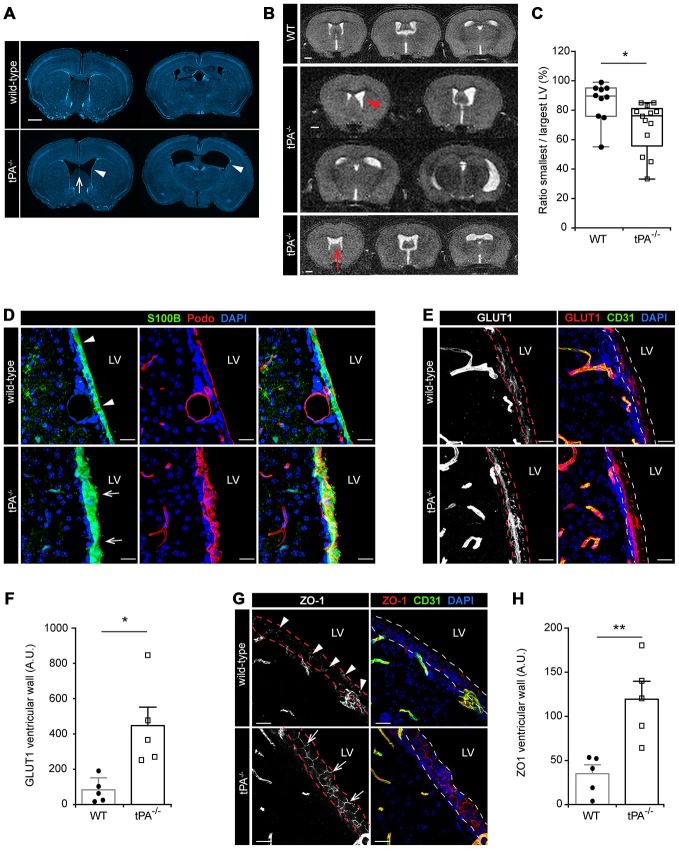
**Lateral ventricular defects and abnormal ependymal lining in the brains of tPA^−/−^ mice.** Lateral ventricular abnormalities (arrowheads) and hypoplastic septum separating the lateral ventricles (LV; arrows) were seen in *tPA^−/−^* mice in **(A)** *ex vivo* sections visualized with DAPI and with **(B)** *in vivo* magnetic resonance imaging (MRI) of WT and *tPA*^−/−^ adult mice. **(B)** Montage from MR scans of an adult WT female mouse (upper panel) and two *tPA*^−/−^ mice (middle panels, images from a male adult *tPA*^−/−^ mouse and lower panel, images from a female adult *tPA*^−/−^ mouse). **(C)** Quantification of the ratio between the smallest to the largest ventricular size from the MRI scans confirmed that *tPA*^−/−^ mice (*n* = 13) displayed a significant asymmetry in lateral ventricular size as compared to WT littermate controls (*n* = 10). The quantifications in **(C)** were made using Image J to trace the entire area of the right and left ventricle respectively. **(D–H)** Abnormal ependymal lining of the LV in *tPA*^−/−^ mice were revealed through immunofluorescent stainings and confocal microscopy on brain sections from WT (*n* = 5) and *tPA*^−/−^ (*n* = 5) mice. Analysis of the ependymal cell lining of the lateral wall of the LV was conducted using antibodies against S100B and Podocalyxin (Podo) **(D)**, GLUT1 **(E–F)** and ZO1 **(G–H)**. Note the thicker ependymal lining in *tPA*^−/−^ mice (arrows, **D**) compared to WT littermate controls (arrowheads, **D**) and the punctate pattern of ZO1 staining in WT (arrowheads, **G**) compared to the continuous tight junctions between ependymal cells in *tPA*^−/−^ mice (arrows, **G**). Quantification of ependymal GLUT1 and ZO1 staining (between the dashed lines in **E,G**) by pixel intensity from six (GLUT1) and four (ZO1) maximum intensity confocal *Z*-stacks per animal revealed an significant increase of both GLUT1 **(F)** and ZO1 **(H)** staining in the ventricular wall of *tPA*^−/−^ mice compared to WT littermate controls. The data shown are representative quantifications of two to six maximum intensity confocal *Z*-stacks per animal from two independent staining experiments. The images display **(A)** stitched tiling of epifluorescent images (taken with 10× objective) and **(D,E,G)** maximum intensity projections generated from confocal *Z*-stacks (22 μm). Each picture is a representative from different individuals. Cell nuclei were visualized with DAPI and vessels with podocalyxin (Podo) or CD31. Data presented as **(C)** box-and-whiskers plot for distribution with min to max variance and **F,H**) mean ± SEM. Statistical significance was determined by student’s unpaired *t*-test and *P < 0.05; **P < 0.01 relative to control. Scale bars **(A–B)** 1 mm and **(D,E,G)** 20 μm. LV, Lateral ventricles. Arbitrary units; A.U.

During the characterization of the *tPA^−/−^* mice we noted that these mice presented with asymmetric LV (Figure 6) similar to what we recently reported for *Pdgfc*^−/−^ mice on C57BL/6 background (Fredriksson et al., 2012). Analysis of brain sections from *tPA*^−/−^ mice (*n* = 5) by DAPI staining revealed that the abnormal LV (arrowheads) coincided with a hypoplastic septum, a defect that was not noted in any WT animals (*n* = 5; arrow, Figure 6A). To ensure that the ventricular abnormalities were not artifacts of tissue preparation, *in vivo* MRI in live mice was employed. Figure 6B shows the montage from MRI scans of one adult WT mouse (upper panel) and two *tPA*^−/−^ mice (middle and lower panels). These MR scans illustrate displacement of the septum towards the side of the smaller ventricle in one of the *tPA*^−/−^ brains (arrowhead) and a hypoplastic septum in the other *tPA*^−/−^ mouse (arrow, Figure 6B). This displacement of the septum in the *tPA*^−/−^ mice made the smaller ventricle appear compressed. Comparing the total area of the smallest to the largest ventricle from the MR scans revealed significant asymmetry of the LV (*P* < 0.05) in the *tPA*^−/−^ mice (70 ± 5%, *n* = 13) as compared to WT littermate controls (86 ± 4%, *n* = 10; Figure 6C), thus confirming the observation from the DAPI analysis. The ventricular abnormalities seen in the *tPA*^−/−^ mice appeared to be milder when compared to the asymmetry of the LV reported in *Pdgfc*^−/−^ mice (56 ± 6%; Fredriksson et al., 2012).

In adult mammals, the cerebral ventricles are normally lined by a single layer of uninterrupted ciliated squamous to columnar ependymal cells, which form the interface between the brain parenchyma and the ventricular cavities (Bruni, 1998). Based on the above findings of lateral ventricle abnormalities in the *tPA^−/−^* mice we then examined the integrity of the ependymal cell lining in adult tPA mutant mice. In the *tPA*^−/−^ mice (*n* = 5) we noted that the ependymal lining, here visualized by the ependymal marker S100B (Bruni, 1998), was distorted and that the normal cuboidal shape of ependymal cells was often lost in the *tPA*^−/−^ animals (arrows, Figure 6D). The ependymal lining appeared pleomorphic in *tPA*^−/−^ mice and did not form a uniform single layer as seen in WT littermate controls (*n* = 5; arrowheads, Figure 6D). This was further confirmed by immunostaining with podocalyxin (Podo) showing that the strong apical border staining seen in WT ependyma was abnormal and distorted in *tPA*^−/−^ mice (Figure 6D, middle panels). Unlike what we saw in the* Pdgfc*^−/−^ mice, we did not find any signs of ependymal denudation or decreased expression in glucose transporter 1, GLUT1, (a marker for mature ependyma; Silva-Alvarez et al., 2005) in the ventricular wall of *tPA*^−/−^ mice. On the contrary, our analysis showed an increase in GLUT1 staining in the ventricular wall (indicated by the dashed lines) in *tPA*^−/−^ mice (*n* = 5) compared to WT littermate controls (*n* = 5; Figure 6E). This was confirmed by quantification of the ventricular GLUT1 staining (*P* < 0.05; *tPA*^−/−^ = 580 ± 144% of WT; Figure 6F). Further, the ependymal cells in the ventricular walls normally display a punctate pattern of ZO1 distribution, indicative of discontinuous tight junctions (Petrov et al., 1994). This punctate pattern was seen in the WT littermate controls (arrowheads, Figure 6G), but in *tPA*^−/−^ mice, the ZO1 staining indicated continuous tight junctions between the ependymal cells (arrows, Figure 6G). Quantification of the ZO1 staining showed significantly increased levels of ZO1 (*P* < 0.01) in the ventricular wall of *tPA*^−/−^ mice (*n* = 5) compared to WT littermate controls (*n* = 5; *tPA*^−/−^ = 363 ± 62% of WT; Figure 6H).

Taken together these results demonstrate that ablation of tPA affects the cerebrovascular bed as well as ventricular development and ependymal integrity.

## Discussion

Thrombolytic treatment with tPA only benefits a limited number of patients with ischemic stroke, and the limitations appear to be due in part to unique activities of tPA in the brain beyond its well established role in fibrinolysis (Nicole et al., 2001; Wang et al., 2003; Yepes et al., 2003; Su et al., 2009). Development of improved therapies for stroke will therefore benefit from understanding the unique characteristics of the role that endogenous tPA plays in the CNS. Previous work has demonstrated an effect of tPA as a necessary and sufficient regulator of cerebrovascular permeability and that targeting tPA-induced opening of the BBB improves outcome of disease (Yepes et al., 2003; Su et al., 2008; Fredriksson et al., 2015). To advance our understanding of the role of tPA in regulation of cerebrovascular integrity we performed a detailed characterization of the cerebrovasculature in tPA deficient mice. We demonstrate that tPA ablation leads to reorganization of the vascular bed in the adult murine brain, with a significant decrease in average vessel diameter, mainly due to a reduction in the number of large and medium sized vessels. These vessels are normally enveloped by PDGFRα+ perivascular astrocytes (Fredriksson et al., 2015), and we believe that the loss of these larger vessels in *tPA^−/−^* brains accounts for the observed reduction in vessel-associated PDGFRα expression. This is particularly interesting since our previous work has shown that the effect of tPA in regulation of cerebrovascular permeability is mediated via tPA-catalyzed activation of PDGF-C, and subsequently PDGFRα signaling on perivascular astrocytes in the neurovascular unit (Fredriksson et al., 2004, 2005; Su et al., 2008). The data presented here suggests that the protection of barrier properties seen utilizing these *tPA*^−/−^ mice might be due, at least in part, to the cerebrovascular rearrangements, further supporting our idea that tPA-induced changes in cerebrovascular permeability proceeds dysregulation of neuronal signaling pathways (Fredriksson et al., 2015). In support of the notion, that congenital cerebrovascular rearrangements contribute to increased cerebrovascular integrity in *tPA*^−/−^ mice, is our intriguing observation that ablation of tPA results in increased number of ERG-positive endothelial cells. Overexpression of this endothelial transcription factor *in vivo* has recently been shown to reduce vascular permeability during VEGF-induced angiogenesis (Birdsey et al., 2015). It should be noted however, that tPA is sufficient to directly control cerebrovascular permeability, not only due to congenital rearrangements in the cerebrovascular bed, since intraventricular injection of tPA induces BBB opening in WT mice (Yepes et al., 2003). Further, cerebrovascular permeability induced by experimental ischemic stroke can be blocked by neuroserpin (Yepes et al., 2003), the specific inhibitor of tPA in the brain (Fredriksson et al., 2015).

In addition to its role in regulation of cerebrovascular permeability (Fredriksson et al., 2015), tPA has been implicated in other cerebrovascular responses, including neurovascular coupling (Park et al., 2008; Su et al., 2009). Neurovascular coupling refers to increases in cerebral blood flow in response to neuronal activity and ensures coupling between energy demand and supply (Iadecola, 2004; Hamel, 2006). In the study by Park et al. (2008) they utilized tPA^−/−^ mice to demonstrate that cerebral blood flow in the corresponding barrel cortex of these mice showed a sustained attenuation after whisker stimulation compared with WT mice. Another study has recently demonstrated that neurovascular coupling of local neural activity to local blood flow takes place exclusively at ASMA+ SMC-covered arterioles, but not in pericyte-covered capillaries (Hill et al., 2015). Interestingly, tPA has been shown to be expressed around arterioles in the CNS (Levin and del Zoppo, 1994; Su et al., 2008; Fredriksson et al., 2015). Given the decreased number of large diameter ASMA+ vessels observed in *tPA^−/−^* mice it therefore seems plausible to suggest that the cerebrovascular rearrangements associated with tPA ablation may explain the attenuated neurovascular coupling response in *tPA*^−/−^ mice. Arguing against this are the findings that ectopic administration of tPA to *tPA*^−/−^ mice restored the neurovascular coupling response (Park et al., 2008) and that *in vivo* vasomotion in awake mice occurs in vessels covered by SMCs, regardless of their diameter (Hill et al., 2015). In addition, the add-back experiment (Park et al., 2008) implicates there may be another downstream target of tPA than PDGFRα signaling in the neurovascular coupling response since vessel-associated expression of the receptor is reduced in *tPA*^−/−^ mice.

Our previous findings established a novel, previously unknown, role for PDGF-C/PDGFRα signaling in cerebroventricular development (Fredriksson et al., 2012). This was later confirmed by a study showing that aberrant PDGFRα signaling in primary cilia, and a subsequent loss of NG2(+)PDGFRα(+) neural progenitor cells in the subventricular zone, is associated with ventricular malformations (Carter et al., 2012). The findings that *tPA^−/−^* mice displayed mild cerebroventricular malformations is especially intriguing since this provides an *in vivo* link between tPA and PDGF-C signaling during CNS development. As reported for the PDGF-C deficient (*Pdgfc*^−/−^) mice (Fredriksson et al., 2012), where the smaller ventricle appeared to arise from displacement of the septum, i.e., the structure separating the LV, this also seemed to be the case in *tPA*^−/−^ mice. The overall effect on cerebroventricular assymmetry seemed less affected by ablation of tPA compared to PDGF-C ablation. Our analysis did however identify a few cases of severe assymetry and incomplete development of the septum, a feature reminiscent of cavum septum pellucidum in humans. A cavum of the septum is often present at birth but eliminated as the septal leaves fuse postnatally (Raine et al., 2010) and preservation of the cavum in adults has been suggested to be due to underdevelopment of limbic structures such as the hippocampus, amygdala and septal nuclei (Nopoulos et al., 2000; Kim et al., 2007), places where high expression of tPA is seen. Contrary to our earlier study, showing that PDGF-C ablation causes defects in the ependymal lining of the LV, including ependymal denudation and reduced expression of GLUT1 in the ventricular wall (Fredriksson et al., 2012), we did not find any signs of ependymal denudation in *tPA*^−/−^ mice and instead noted an increase in ventricular expression of GLUT1 and ZO1. This is indicative of a more mature ventricular lining with higher integrity in *tPA*^−/−^ mice compared to WT littermate controls and *Pdgfc*^−/−^ mice. The opposing effects of tPA and PDGF-C ablation on ependymal development, and in cerebrovascular development where *Pdgfc*^−/−^ mice, contrary to *tPA*^−/−^ mice, display a significant overall increase in vessel diameter and ASMA+ expression, suggests additional, independent roles, of the two in cerebral development. Whether the congenital defects observed in *tPA*^−/−^ mice are directly controlled by tPA-mediated processes, or an indirect consequence of tPA ablation, remains to be established. It will therefore be interesting to utilize the *tPA*^−/−^ mice, *Pdgfc*^−/−^ mice (Fredriksson et al., 2012) and conditional PDGFRα knockout mice (Carter et al., 2012) to further unravel aspects and underlying molecular mechanisms of cerebral vascular, ventricular and ependymal development.

In conclusion, our data demonstrate that tPA plays an important role in normal cerebral vascularization and normal cerebral ventricular formation. Thus, this study enhances our understanding of the role of tPA in the CNS, in particular the role in regulation of cerebrovascular integrity, and might help explain how vascular barrier defects contribute to stroke evolution. In addition, since increased cerebrovascular permeability constitutes a significant pathologic factor in the development of other neurologic diseases, including seizures (Friedman et al., 2009) and traumatic brain injury (Shlosberg et al., 2010), and since tPA-mediated PDGFRα signaling has been implicated in regulation of vascular integrity in these diseases (Fredriksson et al., 2015; Su et al., 2015), our findings might be of importance not only for stroke but also for unrelated CNS disorders where vascular integrity is compromised.

## Conflict of Interest Statement

The authors declare that the research was conducted in the absence of any commercial or financial relationships that could be construed as a potential conflict of interest.
