# Supplementary material for: tPA Deficiency in Mice Leads to Rearrangement in the Cerebrovascular Tree and Cerebroventricular Malformations
Source: Front Cell Neurosci. 2015 Nov 30;9:456. doi: 10.3389/fncel.2015.00456 (PMC4663266; doi:10.3389/fncel.2015.00456)
Supplement: Supplementary file 1 [file Table_1.pdf]

All observations from **5** tPA ko mice and **5** littermate controls at bregma -1.5 (except 6F and 6H, bregma +1). Stainings performed free float on 50 µm vibratome sections.

Fig	Stain	Staining repeated (# of times)	No. of images analyzed/ animal/ staining (brain region)	Area (X*Y*Zµm)	Analyzed (ImgJ, Volocity, Photoshop)	Results
**1B**	CD31 (diameter)	4 independent times (I – IV)	I. 9 (3CTX,2HPF,2TH,2Am)<br>II. 4 (2CTX,2HPF)<br>III. 4 (2CTX,2TH)<br>IV. 4 (2CTX,2HPF)	I. 320x320x22µm<br>II. 160x160x22µm<br>III. 160x160x22µm<br>IV. 160x160x22µm	I. Volocity+ImgJ<br>II. Volocity+ImgJ<br>III. ImgJ<br>IV. ImgJ	**I.** WT=8.20um KO=5.27um, *P*<0.001 (# of vessels measured WT=689, KO=774)<br>**II.** WT=5.32um KO=4.44um, *P*=0.002 (# of vessels measured WT=229, KO=238)<br>**III.** WT=5.83um KO=4.48um, *P*=0.02 (# of vessels measured WT=213, KO=237)<br>**IV.** WT=6.03um KO=4.75um, *P*=0.005 (# of vessels measured WT=211, KO=269)<br>Combined WT=6.34um KO=4.73um, *P*=0.04
**1C**	CD31 (size distrib.)	4 independent times (I – IV)	I. 9 (3CTX,2HPF,2TH,2Am)<br>II. 4 (2CTX,2HPF)<br>III. 4 (2CTX,2TH)<br>IV. 4 (2CTX,2HPF)	I. 320x320x22µm<br>II. 160x160x22µm<br>III. 160x160x22µm<br>IV. 160x160x22µm	I. Volocity+ImgJ<br>II. Volocity+ImgJ<br>III. ImgJ<br>IV. ImgJ	>10um/5-10um/<5um<br>**I.** WT=9%/74%/17% KO=2%/38%/60% (# of vessels measured WT=689, KO=774)<br>**II.** WT=3%/45%/52% KO=1%/24%/75% (# of vessels measured WT=229, KO=238)<br>**III.** WT=10%/42%/48% KO=1%/25%/74% (# of vessels measured WT=213, KO=237)<br>**IV.** WT=10%/36%/54% KO=1%/32%/67% (# of vessels measured WT=211, KO=269)<br>Combined *P*=0.008 /0.08/ 0.03 WT=8%/49%/43% KO=1%/30%/69%
**1D**	CD31 (intensity)	4 independent times (I – IV)	I. 9 (3CTX,2HPF,2TH,2Am)<br>II. 4 (2CTX,2HPF)<br>III. 4 (2CTX,2TH)<br>IV. 4 (2CTX,2HPF)	I. 320x320x22µm<br>II. 160x160x22µm<br>III. 160x160x22µm<br>IV. 160x160x22µm	I. Volocity+ImgJ<br>II. Volocity+ImgJ<br>III. Volocity+ImgJ<br>IV. Volocity	**I.** KO = 49% of WT, P = ns<br>**II.** KO = 100% of WT, P = ns<br>**III.** KO = 70% of WT, P = ns<br>**IV.** KO = 80% of WT, P = ns<br>Combined KO=75% of WT, *P* = ns

CTX=cortex, HPF=hippocampus, TH=Thalamus, CP=Caudate Putamen, HY=Hypothalamus, Am=Amygdala, LV=Lateral Ventricles

Fig	Stain	Staining repeated (# of times)	No. of images analyzed/ animal/ staining (brain region)	Area (X*Y*Zµm)	Analyzed (ImgJ, Volocity, Photoshop)	Result
**1E**	Podo (intensity)	4 independent times (I – IV)	I. 7 (3CTX,4HPF)<br>IIa. 8 (3CTX,1HPF,3CP,1Am)<br>IIb. 4 (2CTX,2HPF)<br>III. 9 (4CTX,2HPF,2Am,1TH)<br>IV. 11 (3CTX,2HPF,2TH,2CP,2Am)	I. 640x640x4µm<br>IIa. 1280x1280x15µm<br>IIb. 160x160x22µm<br>III. 640x640x22µm<br>IV. 1280x1280x20µm	I. ImgJ<br>IIa. Volocity+ImgJ<br>IIb. Volocity+ImgJ<br>III. ImgJ<br>IV. Volocity+ImgJ	**I**. KO = 106% of WT, *P* = ns<br>**IIa**. KO = 126% of WT, *P* = ns<br>**IIb**. KO = 119% of WT, *P* = ns<br>**III**. KO = 105% of WT, *P* = ns<br>**IV**. KO = 127% of WT, *P* = ns<br>Combined KO=116% of WT, *P* = ns Analysis of the Podocalyxin staining shows similar size redistribution in the cerebrovascular tree of tPA ko mice compared to WT as analysis of CD31 stain
**2B**	ERG1 (number of positive cells)	2 independent times (I – II)	Ia. 9 (4CTX,2HPF,2Am,1TH)<br>Ib. 5 (2CTX,2Am,1TH)<br>IIa. 4 (2CTX,2HPF)<br>IIb. 5 (2CTX,2Am,1TH)	Ia. 160x160x22µm<br>Ib. 320x320x18µm<br>IIa. 160x160x22µm<br>IIb. 320x320x18µm	Ia. Volocity+ImgJ<br>Ib. Volocity+ImgJ<br>IIa. Volocity+ImgJ<br>IIb. Volocity+ImgJ	**Ia**. KO = 150% of WT, *P* < 0.01 (Avg # per img WT=12, KO=18)<br>**Ib**. KO = 133% of WT, *P* = 0.04 (Avg # per img WT=80, KO=107)<br>**IIa**. KO = 133% of WT, *P* < 0.01 (Avg # per img WT=15, KO=20)<br>**IIb**. KO = 131% of WT, *P* = 0.04 (Avg # per img WT=71, KO=93)<br>Combined KO=137% of WT, *P* = 0.04
**2C**	ERG1 (normalized to CD31)	2 independent times (I – II)	Ia. 9 (4CTX,2HPF,2Am,1TH)<br>IIa. 4 (2CTX,2HPF)	Ia. 160x160x22µm<br>IIa. 160x160x22µm	Ia. Volocity+ImgJ<br>IIa. Volocity+ImgJ	**Ia**. 2x more ERG/CD31 in KO compared to WT, *P* < 0.001<br>**IIa.** 1.5x more ERG/CD31 in KO compared to WT, *P* = 0.003<br>Combined KO=1.75x of WT, *P* = 0.01

CTX=cortex, HPF=hippocampus, TH=Thalamus, CP=Caudate Putamen, HY=Hypothalamus, Am=Amygdala, LV=Lateral Ventricles

Fig	Stain	Staining repeated (# of times)	No. of images analyzed/ animal/ staining (brain region)	Area (X*Y*Zμm)	Analyzed (ImgJ, Volocity, Photoshop)	Result
**3B**	ASMA (intensity)	3 independent times (I – III)	I. 11 (3CTX,2HPF,2TH,2CP,2Am)<br>II. 11 (3CTX,2HPF,2TH,2CP,2Am)<br>IIIa. 8 (3CTX,1HPF,3CP,1Am)<br>IIIb. 4 (2CTX,2HPF)	I. 1280x1280x20μm<br>II. 1280x1280x20μm<br>IIIa. 1280x1280x15μm<br>IIIb. 160x160x22μm	I. Volocity+ImgJ<br>II. Volocity+ImgJ<br>IIIa. Volocity+ImgJ<br>IIIb. Volocity	**I.** KO = 99% of WT, *P* = ns<br>**II.** KO = 83% of WT, *P* = ns<br>**IIIa**. KO = 79% of WT, *P* = ns<br>**IIIb**. KO = 111% of WT, *P* = ns<br>Combined KO=93% of WT, *P* = ns
**3C**	ASMA (diameter)	3 independent times (I – III)	I. 11 (3CTX,2HPF,2TH,2CP,2Am)<br>II. 11 (3CTX,2HPF,2TH,2CP,2Am)<br>IIIa. 8 (3CTX,1HPF,3CP,1Am)	I. 1280x1280x20μm<br>II. 1280x1280x20μm<br>IIIa. 1280x1280x15μm	I. Volocity+ImgJ<br>II. Volocity+ImgJ<br>IIIa. Volocity+ImgJ	**I.** WT=24.2um KO=16.6um (67% of WT) *P*=0.001. (WT 422/KO 558 vessels)<br>**II.** WT= 22.9um KO= 15.4um (66% of WT). *P*=0.001 (WT 404/KO 640 vessels)<br>**IIIa.** WT=17.8um KO=14.1um (79% of WT), *P*=0.07. (WT 152/KO 164 vessels)<br>Combined WT=21.6um KO=15.3um, *P*=0.04
**3D**	ASMA (size distrib.)	3 independent times (I – III)	I. 11 (3CTX,2HPF,2TH,2CP,2Am)<br>II. 11 (3CTX,2HPF,2TH,2CP,2Am)<br>IIIa. 8 (3CTX,1HPF,3CP,1Am)	I. 1280x1280x20μm<br>II. 1280x1280x20μm<br>IIIa. 1280x1280x15μm	I. Volocity+ImgJ<br>II. Volocity+ImgJ<br>IIIa. Volocity+ImgJ	>30um/15-30um/<15um<br>**I.**<br>WT=22%/66%/12%, KO=5%/47%/48%, (# of vessels WT 422/KO 558)<br>**II.**<br>WT=19%/65%/17%, KO=5%/34%/61%, (# of vessels WT 404/KO 640)<br>**IIIa.**<br>WT=5%/47%/48%, KO=1%/31%/68% (# of vessels WT 152/KO 164)<br>Combined *P*=0.05/ 0.04/ 0.04<br>WT=15%/59%/26% KO=4%/37%/59%

CTX=cortex, HPF=hippocampus, TH=Thalamus, CP=Caudate Putamen, HY=Hypothalamus, Am=Amygdala, LV=Lateral Ventricles

Fig	Stain	Staining repeated (# of times)	No. of images analyzed/ animal/ staining (brain region)	Area (X*Y*Zµm)	Analyzed (ImgJ, Volocity, Photoshop)	Result
**3F**	CD13 (intensity)	3 independent times (I – III)	Ia. 9 (4CTX,2HPF,2Am,1TH)<br>Ib. 4 (2CTX,2HPF)<br>II. 9 (4CTX,2HPF,2Am,1TH)<br>III. 4 (2CTX,2HPF)	Ia. 640x640x22µm<br>Ib. 160x160x22µm<br>II. 640x640x22µm<br>III. 160x160x22µm	Ia. Volocity+ImgJ<br>Ib. Volocity+ImgJ<br>II. Volocity+ImgJ<br>III. Volocity+ImgJ	**Ia**. KO = 70% of WT, *P* = 0.03<br>**Ib**. KO = 71% of WT, *P* = ns<br>**II**. KO = 91% of WT, *P* = ns<br>**III**. KO = 80% of WT, *P* = ns<br>Combined 78% of WT, *P* = 0.02
**5B**	PDGFRα (intensity)	3 independent times (I – III)	I. 9 (3CTX,2HPF,2TH,2Am)<br>II. 5 (2CTX,1HPF+TH,2Am)<br>III. 5 (2CTX,1HPF+TH,2Am)	I. 320x320x22µm<br>II. 1500x1500µm (epifluo microscope)<br>III. 1500x1500µm (epifluo microscope)	I. Volocity+ImgJ<br>II. Volocity<br>III. Volocity	**I**. KO = 71% of WT, *P* = ns<br>**II**. KO = 97% of WT, *P* = ns<br>**III**. KO = 82% of WT, *P* = ns<br>Combined KO = 86% of WT, *P* = ns
**5C**	PDGFRα (area of PDGFRα immunoreactivity associated with Podo)	3 independent times (I – III)	I. 9 (3CTX,2HPF,2TH,2Am)<br>II. Entire brain<br>III. Entire brain	I. 320x320x22µm<br>II. 7.2x10.1mm (153 tiles taken w. epifluo at 10x magnification)<br>III. 7.2x10.1mm (153 tiles taken w. epifluo at 10x magnification)	I. ImgJ+PS<br>II. ImgJ+PS<br>III. ImgJ+PS	**I**. KO = 21% of WT, *P* = 0.04 (#vessels WT 133/KO 51, KO=38% of WT)<br>**II**. KO = 30% of WT, *P* = 0.01 (#vessels WT 239/KO 67, KO=28%)<br>**III**. KO = 44% of WT, *P* = 0.008 (#vessels WT 289/KO 110, KO=38%)<br>Combined KO = 32% of WT, *P* = 0.02

CTX=cortex, HPF=hippocampus, TH=Thalamus, CP=Caudate Putamen, HY=Hypothalamus, Am=Amygdala, LV=Lateral Ventricles

Fig	Stain	Staining repeated (# of times)	No. of images analyzed/ animal/ staining (brain region)	Area (X*Y*Zµm)	Analyzed (ImgJ, Volocity, Photoshop)	Result
**6F**	GLUT1 (intensity in ependymal lining)	2 independent times (I – II)	Bregma +1<br>I. 2 (lateral walls of LV)<br>IIa. 2 (lateral walls of LV)<br>IIb. 6 (lateral walls of LV)	I. 160x160x21µm<br>IIa. 200x200x15µm<br>IIb. 160x160x22µm	I. ImgJ<br>IIa. ImgJ<br>IIb. ImgJ	I. KO = 481% of WT, *P* < 0.001<br>IIa. KO = 319% of WT, *P* = 0.03<br>IIb. KO = 580% of WT, *P* = 0.01<br>Combined KO = 460% of WT, *P* = 0.02
**6H**	ZO1 (intensity in ependymal lining)	2 independent times (I – II)	Bregma +1<br>I. 4 (lateral walls of LV)<br>IIa. 2 (lateral walls of LV)<br>IIb. 2 (lateral walls of LV)	I. 160x160x15µm<br>IIa. 200x200x15µm<br>IIb. 160x160x22µm	I. ImgJ<br>IIa. ImgJ<br>IIb. ImgJ	**I.** KO = 363% of WT, *P* = 0.005<br>IIa. KO = 229% of WT, *P* = 0.02<br>IIb. KO = 266% of WT, *P* = 0.03<br>Combined KO = 286% of WT, *P* = 0.04

Supplemental table 1. Summary of stainings, quantifications and results
The table is summarizing the number of independent times each staining has been repeated, the number of images analyzed per brain and staining, in what brain regions the images were taken and the results.
